# Analysis of 363 Genetic Variants in F5 via an Interactive Web Database Reveals New Insights into FV Deficiency and FV Leiden

**DOI:** 10.1055/a-1987-5978

**Published:** 2023-01-09

**Authors:** Christos Efthymiou, Emily H.T. Print, Anna Simmons, Stephen J. Perkins

**Affiliations:** 1 Research Department of Structural and Molecular Biology, University College London, London, United Kingdom

**Keywords:** coagulation factors, protein structure, coagulation, gene mutations

## Abstract

The inherited bleeding disorder Factor V (FV) deficiency and clotting risk factor FV Leiden are associated with genetic variants in the *F5* gene. FV deficiency occurs with mild, moderate, severe, or asymptomatic phenotypes, and either dysfunctional or reduced amounts of plasma FV protein. Here we present an interactive web database containing 363 unique *F5* variants derived from 801 patient records, with 199 FV deficiency-associated variants from 245 patient records. Their occurrence is rationalized based on the 2,224 residue sequence and new FV protein structures. The 199 FV deficiency variants correspond to 26 (13%) mild, 22 (11%) moderate, 49 (25%) severe, 35 (18%) asymptomatic, and 67 (34%) unreported phenotypes. Their variant distributions in the FV domains A1, A2, A3, B, C1 and C2 were 28 (14%), 32 (16%), 34 (17%), 42 (21%), 16 (8%), and 19 variants (10%), respectively, showing that these six regions contain similar proportions of variants. Variants associated with FV deficiency do not cluster near known protein-partner binding sites, thus the molecular mechanism leading to the phenotypes cannot be explained. However, the widespread distribution of FV variants in combination with a high proportion of buried variant residues indicated that FV is susceptible to disruption by small perturbations in its globular structure. Variants located in the disordered B domain also appear to disrupt the FV structure. We discuss how the interactive database provides an online resource that clarifies the clinical understanding of FV deficiency.

## Introduction

**Fig. 1:**
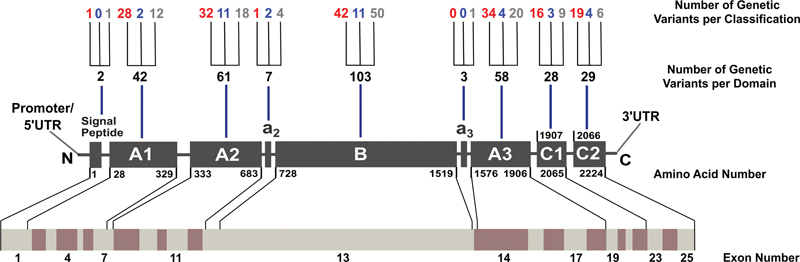
*Distribution of the 363 unique variants identified within the F5 gene and FV protein.* The FV protein is comprised of the A1-A2-a2-B-a3-A3-C1-C2 domains and linkers in that order, and is shown as gray boxes that are not drawn to scale. N and C represent the N- and C-termini, respectively. Residue numbering marks the first and last amino acids which frame each domain, reported in HGVS format (starting with 1 at the signal peptide). The number of variants in each domain is shown above each protein domain. The number of variants associated with FV deficiency ( *red* ), thrombosis or FV Leiden ( *blue* ), or have an unknown association ( *gray* ) are indicated. Intronic variants (19 in total), undefined or not applicable variants (nine), and variants affecting multiple domains (two) are not shown. Below the protein domains, the gene arrangement of 25 exons is shown as alternating light gray and maroon boxes drawn to scale. The FV protein domain to which each exon codes for are indicated. FV, Factor V.

The major disease associated with variants in Factor V (FV) of the blood coagulation cascade is FV deficiency. FV deficiency itself is a bleeding disorder, while FV Leiden is a risk factor for clotting, both of which are associated with genetic variants in the *F5* gene which codes for FV. 1 2 The full-length human *F5* gene has 25 exons (OMIM #612309; Gene ID #2153) spanning approximately 80 kb and is located on chromosome 1 (1q24). 3 The mRNA with 25 exons ( Fig. 1 ) contains 6,672 bp, corresponding to a protein with 2,224 amino acids and a molecular mass of 330 kDa. A 28-residue signal peptide is removed post-translationally in the endoplasmic reticulum, leaving a 2,196 residue protein. FV is comprised of six major domains and two linkers in the order A1-A2-a2-B-a3-A3-C1-C2 ( Fig. 1 ). 4 FV is homologous with coagulation Factor VIII, with their A and C domains having approximately 40% sequence identity. 3 FV is primarily synthesized by the liver whereby it is released into the bloodstream in the inactive zymogenic form. It circulates in the blood and is composed of a heavy chain with domains A1 and A2, and a light chain with domains A3, C1, and C2. 5

FV is a plasma glycoprotein that plays a key role in the coagulation cascade. Specifically, activated FV (FVa) is essential in the prothrombinase complex and thrombin amplification. This complex cleaves prothrombin into thrombin, aiding formation of the platelet plug that is critical in coagulation. FV can be activated either by activated Factor X (FXa) or thrombin. Activation results in the cleavage of FV at R709, R1018, and R1545 (legacy numbering R681, R990, R1517) to release the B domain from the middle of FV. The FV heavy and light chains remain associated through a single calcium ion. Cleavage induces a conformational change, enabling FVa to be a cofactor for FXa to cleave prothrombin. FXa alone has minimal affinity for the substrate prothrombin. However, once activated, FXa complexes with its cofactor FVa to form the prothrombinase complex which has high prothrombin affinity. Prothrombin binds an exosite on the protease complex and is cleaved at two distinct sites, Arg271 and Arg320. Thrombin is subsequently released and facilitates platelet plug formation, preventing excess blood loss. A negative feedback loop controls this process, where thrombin induces the generation of negative control proteins to prevent excess blood clotting. Activated protein C (APC) degrades FVa, thereby preventing additional conversion of prothrombin to thrombin. 3 6

Genetic variants within the *F5* gene are predominantly associated with FV deficiency, for which we report 199 variants. From a clinical point of view, it is considered an autosomal recessive disease. FV deficiency can be classified as Type I or Type II. Type I deficiency is quantitative and defined by low coagulant activity (FV:C) and antigen levels (FV:A). Type II deficiency is qualitative and characterized by low FV:C even with normal FV:A. 7 FV:C in normal human plasma ranges from 60 to 140 IU/dL. 8 The phenotypic classification is determined by the FV:C level with severely, moderately, and mildly deficient individuals having FV:C levels of <1, 1 to 5, and 6 to 10% of normal. 9 Patients with FV deficiency suffer from reduced coagulation and an increased risk of uncontrolled bleeding due to reduced FV:C levels. Individuals with severe FV deficiency are typically homozygous or compound heterozygous for causative mutations, whereas individuals who are partially deficient are heterozygous, with one mutated allele. In contrast, FV Leiden disorders are less frequent, for which we report 37 variants. FV Leiden thrombophilia is linked with APC resistance, whereby FV variants render it less susceptible to inactivation by APC. In particular, APC cleaves FVa at Arg residues R334, R534, R707 (legacy R306, R506, and R679) to inactivate it; mutations in these residues resist inactivation by APC. 10 This results in higher-than-normal coagulation due to increased thrombin production. 11

Individuals with FV deficiency experience variable bleeding symptoms, the severity correlating with the level of protein activity. Individuals with lower FV:C will experience more severe symptoms than those with higher protein activity. Symptom onset varies, though severely affected individuals suffer from an earlier age. Mildly affected individuals experience minor symptoms such as easy bruising, nosebleeds, gum bleeding, and hematuria. Such individuals are also prone to prolonged and excessive postoperative bleeding. Severely affected individuals suffer from more severe bleeds including hemarthrosis, intramuscular bleeds, and hematomas. Additionally, such individuals are more susceptible to melena, bleeding in the gastrointestinal tract, and intracranial hemorrhages. Females suffering from FV deficiency often experience menorrhagia. Such individuals are also at greater risk of pregnancy complications including miscarriage and post-partum hemorrhage. Current treatments include antifibrinolytics, fibrin glue, and fresh frozen plasma which promote blood clotting and reduce bleeding. 12 Those with FV Leiden often do not experience symptoms. However, this polymorphism is associated with a weak risk for venous thrombosis. Symptoms depend on the location of blood clot. In contrast to FV deficiency, an FV Leiden patient who suffers from a vein thrombosis usually needs anticoagulants (more frequently now direct oral anticoagulants rather than coumarins). 8

The development of the first interactive searchable web databases for genetic variants in the coagulation proteases Factor XI https://www.factorxi.org/ in 2003 and Factor IX https://www.factorix.org/ in 2013 paved the way for a more powerful clinical tool to analyze FV variants. These interactive databases allow variants to be assessed alongside the gene and protein sequences, clinical phenotypes, and known three-dimensional protein structures. The Factor XI website was recently upgraded, 13 and a new one developed for the coagulation protease Factor X https://www.factorx-db.org/ . 14 Based on these, we now report to our knowledge the first interactive database for FV variants. An earlier FV database is no longer available. 15 Genetic repositories containing lists of variants include the Genome Aggregation Database (gnomAD; https://gnomad.broadinstitute.org/ 16 ), the Leiden Open-source Variation database (LOVD; http://www.lovd.nl/3.0/home 17 ), the Expert Protein Analysis system (ExPASy; https://www.expasy.org/ 18 ), the ClinVar resource ( http://www.ncbi.nlm.nih.gov/clinvar/ 19 ), and the public release of the Human Gene Mutation Database (HGMD; http://www.hgmd.cf.ac.uk/ac/index.php 20 ). However, as the number of known variants has increased, simple listings of FV variants are impractical and overwhelming. Therefore, an interactive database for FV variants is needed.

**Fig. 2:**
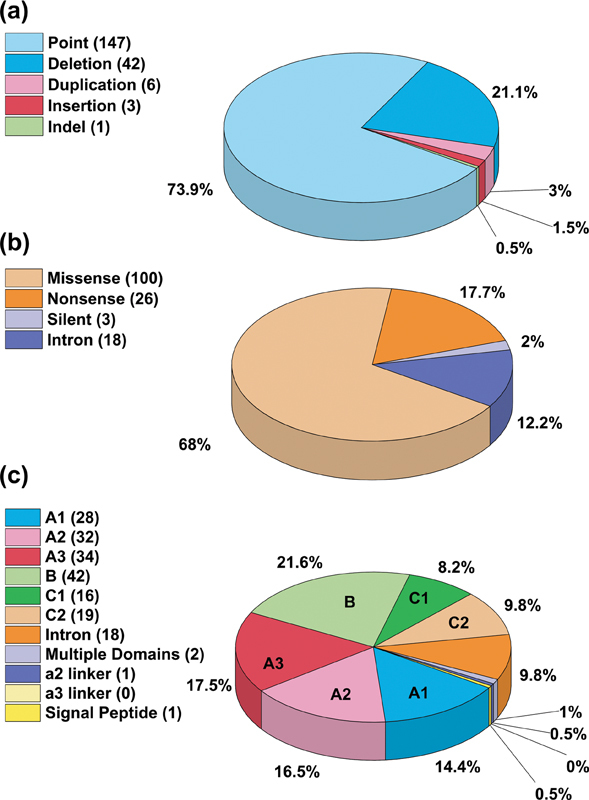
*Distribution of the 199 unique FV deficiency variants found in the F5 gene.* The panels ( **a–c** ) indicate breakdowns of the 199 FV deficiency variants into variant type, effect, and location within the *F5* gene sequence. ( **a** ) The relative frequency of six distinct types of unique variants in the *F5* gene. ( **b** ) Effect of the 147 point variants found in the *F5* gene sequence. ( **c** ) Distribution of the 199 FV variants across the *F5* gene and FV protein domains, although six undefined or not applicable variants are not shown. FV, Factor V.

For the new FV database, we identified 363 unique FV variants, 199 being associated with FV deficiency, and the remainder being associated with FV Leiden or having an unknown association ( Fig. 2 ). F5 variants suggested to have a prothrombotic phenotype are collectively referred as FV Leiden. Our database also includes recent cryo-EM structures that reveal the molecular structures of the five globular A and C domains in FV and FVa for the first time (PDB IDs: 7KVE, 7KXY). 21 In addition, a full-length protein model for FV that includes the disordered B domain structure was predicted by the AlphaFold neural networking and machine learning method. 22 The assembly of these datasets provides insights on the disease mechanisms for FV deficiency. We discuss the mild, moderate, severe, and asymptomatic phenotypes, and demonstrate that disease-associated missense variants were distributed evenly across the globular/disordered and functional/non-functional regions of FV. Therefore, the majority of the variants affect the structural integrity of FV and its protein folding. Clinicians and researchers can use the database as a tool to clarify the significance of the FV variants in FV deficiency.

## Methods

### Source of the FV Database

A total of 363 unique *F5* variants were identified for the interactive FV web database ( https://www.factorv-db.org ) from 801 patients. There was a focus on 199 FV deficiency-associated variants from 245 patient records. The FV database was formatted to follow the layout of our previous FIX, FX, and FXI databases ( https://www.factorix.org ; https://www.factorx-db.org/ ; https://www.factorxi.org ;). 13 14 23 Copyright of the FV website is retained by S.J. Perkins and University College London, and database copying is not permitted without explicit permission. The 363 FV genetic variants were derived from online literature searches of peer-reviewed articles, primarily those in PubMed (https://pubmed.ncbi.nlm.nih.gov/) and the ClinVar resource ( http://www.ncbi.nlm.nih.gov/clinvar/ ). The cut-off date was December 2021 for the current database, and the sources are listed in the “Reference” tab of the FV web database. For the FV web database, data for each variant were compiled into an excel spreadsheet, which was then used to establish the FV MySQL database using phpMyAdmin software ( https://www.phpmyadmin.net/ ) as an intermediary platform. The database is maintained on a University College London server and utilizes HTML, PHP, and JavaScript programming to enable public access via the web. If required for personal or private research use only, a list of the FV variants and their associated fields can be downloaded from the “Variants” menu on the Web site ( Supplementary Materials S1 and S2 ).

### Analysis of the FV Variants

The FV database records DNA and protein changes in the Human Genome Variation Society (HGVS) format ( http://varnomen.hgvs.org/ ). 24 For DNA changes, +1 refers to the A of the ATG initiation codon at the start of the 28-residue pre-leader signal peptide. For protein changes, +1 refers to the ATG initiation codon. In older publications, legacy numbering was used. For these publications, the legacy numbering for reported amino acid changes was converted to HGVS format by adding 28 to the original legacy number (+1 in legacy numbering refers to the first codon of the mature inactivated FV protein and corresponds to +29 in HGVS numbering). Nine human FV and FVa crystal and cryo-EM structures are available in the Protein Data Bank (PDB) at https://www.rcsb.org and these are listed on the FV website. Of these nine structures, six crystal structures are only for partial FV structures. Only the three cryo-EM structures show all five main A1-A3 and C1-C2 domains together (PDB IDs: 7KVE [FV with a resolution of 0.33 nm], 7KVF [FV with a resolution of 0.36 nm], and 7KXY [FVa with a resolution of 0.44 nm]). The 7KVE structure was used for variant analyses in this study as it has the highest resolution. However, no FV structures exist in which the large B domain is resolved as the B domain structure is structurally disordered and cannot be observed even with cryo-EM. 21 To perform the structural analysis of the disordered B domain, an AlphaFold-modeled FV structure was used (https://alphafold.ebi.ac.uk/entry/P12259). AlphaFold is an artificial intelligence system based on neural networks that predicts protein structures based on their amino acid sequence. 22

The Definition of Secondary Structure of Proteins (DSSP) tool (https://www3.cmbi.umcn.nl/xssp/) was used to identify the secondary structure of each residue in the FV cryo-EM structure (PDB ID: 7KVE). 25 Residues that were not visible in the cryo-EM structure were replaced by the AlphaFold structure prediction. Residues were individually assigned secondary structures to be one of either H (α-helix), B (β-bridge), E (extended β-strand), G (310 helix), I (π-helix), T (hydrogen-bonded turn), S (bend) or C (undefined coil region). DSSP was also used to determine the exposed surface areas of each residue of the FV structure in Å ^2^ . The accessibilities were converted into percent accessibility by dividing the DSSP output by the theoretical solvent accessible surface area of the amino acid sidechain in question. 25 26 27 The results were simplified as follows. Percentage accessibilities of 0 to 9% were given the value 0, 10 to 19% the value 1, 20 to 29% the value 2, and so on. Residues with accessibilities of 0 or 1 were classified as buried and those with accessibilities of 2 to 9 were classified as solvent exposed.

To assess the disruptive effect of a given variant on the FV protein structure, four independent substitution analyses were performed on the FV missense variants. These were Polymorphism Phenotyping v2 (PolyPhen-2) http://genetics.bwh.harvard.edu/pph2/ ; Sorting Intolerant From Tolerant (SIFT) https://sift.bii.a-star.edu.sg/www/SIFT_seq_submit2.html ; Protein Variation Effect Analyzer (PROVEAN) http://provean.jcvi.org/seq_submit.php ; and Grantham analysis, using the Grantham matrix from 1974. 28 29 30 31 Both the SIFT and PolyPhen-2 algorithms give variant scores ranging from 0.0 to 1.0. For the PolyPhen-2 algorithm, prediction scores closer to 1.0 indicate variants that are more likely to be damaging. The opposite is true for SIFT where prediction scores closer to 0.0 indicate variants that are more likely to be damaging. Unlike SIFT and PolyPhen-2, the PROVEAN algorithm generates prediction scores that are valued against a threshold; variants with scores above −2.5 are considered neutral and those below −2.5 deleterious. Lastly, the Grantham analysis differs from the other three because its prediction scores are not sequence specific but are instead based on the amino acids which were substituted. Grantham scores range from 0 to 215, with a larger score indicating a variant substitution that is more likely to be damaging. Variants with a Grantham score of 0 are silent and have no effect on protein structure and function.

## Results

### Classification of FV Deficiency Variants in the Updated Interactive Web Database

The interactive FV web database ( https://www.factorv-db.org ) presents information regarding 363 unique genetic variants ( Fig. 2 ) from 801 patient entries. The variants were identified from 135 research articles in PubMed ( https://www.ncbi.nlm.nih.gov/pmc/ ) and the ClinVar resource ( https://www.ncbi.nlm.nih.gov/clinvar/ ) that presented another 123 unique variants or 34% of the total. The database home page features two movies of the monomeric six-domain FV AlphaFold predicted structure and the five-domain FV cryo-EM structure, both of which visualized the spatial distribution of the variants. Users can perform simple searches on the website by amino acid or nucleotide number, or perform an advanced search based on several criteria including variant type, effect, domain location, reference, etc. The database has interactive capabilities derived from our recently upgraded FIX and FX websites ( https://www.factorix.org ; https://www.factorx_db.org ), including a site map which facilitates user navigation. 13 14 23 32 The site map displays the entire FV amino acid sequence, this being highlighted to mark the boundaries of domains and linkers ( Fig. 1 ). Any known missense variants are linked in the sequence and accessed by clicking on the corresponding amino acid. The genome aggregation database (gnomAD) version 2.1.1 (https://gnomad.broadinstitute.org) was used to provide allelic frequencies (AF) for FV variants in the database when possible. The gnomAD v2.1.1 dataset included 125,748 exome sequences and 15,708 whole-genome sequences. Thus 179 (49.3%) of the 363 identified FV variants were found in gnomAD. The AF indicates the relative frequency of a variant at a specific genetic location. Using an AF cut-off of 0.01, an AF> 0.01 indicated a commonly occurring variant, and only 21 of the 179 showed an AF> 0.01. The remaining 158 variants showed an AF <0.01, underscoring that most FV variants are rare ones. Using a stricter AF cut-off of 0.001, only 13 additional variants occurred more frequently than this, leaving 145 FV variants with known AF as rare ones from gnomAD. Additional information is provided for missense variants through the Polyphen-2, SIFT, PROVEAN, and Grantham scores (Methods), all of which predict if the variant is damaging to FV or benign. Other features include a multiple sequence alignment of human FV with FV from other species, helping users to understand the phylogenetic history of the *F5* gene and the extent of residue conservation in related sequences for each variant. Java applets must be enabled within the browser to permit the mutation map, the multiple sequence alignment, and other related features to be seen. An interactive FV structure is presented in a Jmol viewer onto which missense variants can be mapped and viewed for a clearer structural and functional assessment. Users are able to switch between two cryo-EM structures and the AlphaFold full-length protein model. Lastly, all nine currently known FV structures and their literature references are listed on the website to provide an up-to-date knowledge of FV structural research.

Compared with the FV deficiency variants in the database, there were fewer FV Leiden/thrombosis variants, thus a database comparison between these two sets of variants was not straightforward. In addition, the ClinVar variants in the database did not mention the associated disorder. Accordingly, we focus only on the 199 FV deficiency-associated variants. These were classified using the residual FV activity (FV:C) into asymptomatic (>10%), mild (6–10%), moderate (1–5%), and severe (<1%) disease based on a normal activity range of 60 to 140 IU/dL. Of the 199 variants, 26 (13%) were mild, 22 (11%) were moderate, 49 (25%) were severe, 35 (18%) were asymptomatic, and 67 (34%) were unreported phenotypes. Classifying by the type of genetic change, point variants comprised 73.9%, deletions 21.1%, duplications 3.0%, insertions 1.5%, and indels 0.5% ( Fig. 2a ). Point variants can be classified into missense, nonsense, silent, and intronic variants, corresponding to 68.0, 17.7, 2.0, and 12.2% of point variants, respectively ( Fig. 2b ). The 199 unique variants were distributed throughout the *F5* gene and FV protein structure, with variants found in all six A, B, and C domains, as well as the a2 linker ( Fig. 2c ). Point variants found in more than five patients occurred more often in the A and C domains compared with the disordered B domain (green, Supplementary Fig. S1 ).

The distribution of amino acid variants in FV was assessed by a substitution analysis of the 100 FV deficiency missense variants. Only single nucleotide mutations were observed. The dark greyed boxes of Supplementary Fig. S2 signify two nucleotide changes for a given variant, of which there were none in *F5* . The total count for each missense change showed that Arg (18), Ser (11), Cys(9), Asp(7), and Leu (6) were the five most commonly affected residues (right column, Supplementary Fig. S2 ). The most common resulting substitution was Gly (15) (bottom row, Supplementary Fig. S2 ). The predominance of positively charged Arg and negatively charged Asp missense variants suggested that ionic interactions were important for the correct function of FV. The prevalence of the polar uncharged residue Ser and the hydrophobic residues Leu and Val suggested that the internal FV globular protein structure had been disturbed by protein misfolding mutations. Cys residues are important for forming two disulfide bridges in each of the A1 and A2 domains for stabilization, and likewise one disulfide bridge in each of the A3, C1, and C2 domains, explaining the prevalence of Cys residue mutations ( Supplementary Fig. S1 ).

### Cryo-EM and AlphaFold Structure Analysis of Secondary Structures

**Fig. 3:**
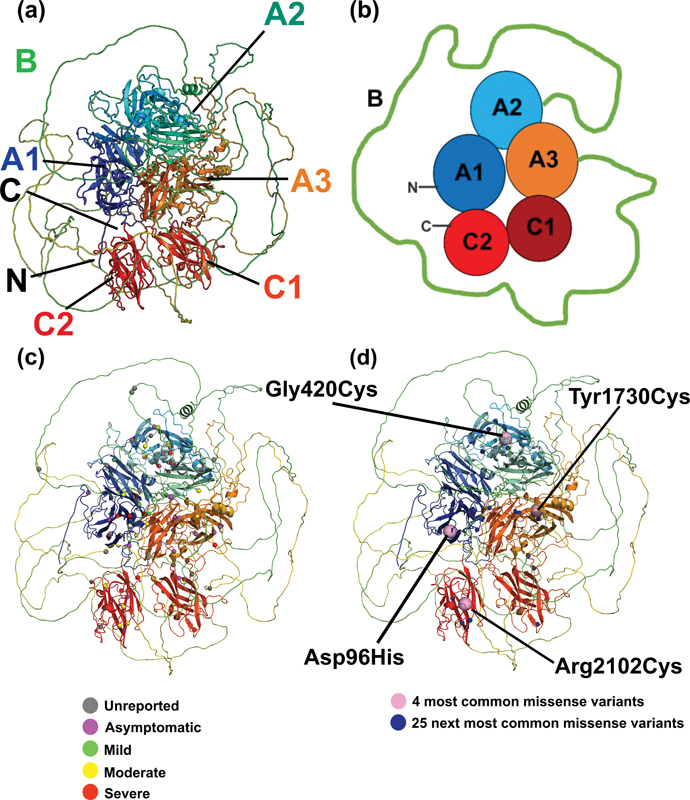
*Structural and schematic views of FV deficiency variants within the FV domains.* ( **a** ) The full FV structure is shown in ribbon format from the AlphaFold prediction. The structure is shown in rainbow colors, with blue corresponding to the N-terminal region and red corresponding to the C-terminal region. The N-terminus and C-terminus are denoted by N and C, respectively. ( **b** ) The FV structure from ( **a** ) is shown schematically in cartoon form in the same orientation and colors. The globular A1, A2, A3, C1, and C2 domains are denoted by filled circles. The disordered B domain is schematically represented by a green line. ( **c** ) The 100 missense variants are mapped to the ribbon diagram, where the phenotype classifications of mild, moderate, and severe effects are denoted as the traffic light colors of green, yellow, and red, respectively. Missense variants with unreported phenotype are shown in gray and asymptomatic cases are shown in purple. ( **d** ) The 25 most commonly reported variants are shown as spheres in the ribbon structure of FV shown in ( **a** ). Blue spheres denote the fifth to twenty-fifth more common variants, and the four magenta spheres denote the four most common variants in FV ( Supplementary Fig. S3 below). FV, Factor V.

The FV protein structure enabled the three-dimensional distribution of the variants to be visualized and clarified the molecular basis of FV deficiency. In our database, three molecular structures were used for structural analysis of genetic variants. These included the two new cryo-EM structures for FV and FVa (PDB IDs: 7KVE and 7KXY, respectively), both of which lack the disordered B domain, and the AlphaFold full-length model of FV that includes a model of the B domain. 21 22 When the FV structure (7KVE) was subjected to Ramachandran plot quality analysis ( http://molprobity.biochem.duke.edu 33 ), 81.4% (1115 residues) of amino acids were categorized in the “most favored” conformational regions, 18.3% (251) were in the “additional allowed” regions, and 0.3% (5) were conformational outliers. This outcome was superior when compared with the FVa (7KXY) structure for which the corresponding figures were 69.8, 26, and 4.2% (805, 300, and 48 residues out of a total of 1,153), respectively. The AlphaFold prediction for full-length FV was based on new structural methods for accurate predictions using artificial intelligence. 21 22 Although AlphaFold is an improvement on previous structural prediction methods, it is difficult to assess the quality of outcome. However, AlphaFold provides a score for each residue called the predicted local-distance difference test (pLDDT), which measures the local reliability for each residue from 0 to 100. For FV, the majority of the residues in the five globular domains have scores above 70, indicating high confidence in the prediction. However, nearly all of the B domain residues have scores below 50, indicating a low confidence in the prediction. This is expected because the AlphaFold confidence scores are correlated with the existence of homologs in the PDB. 22 34 Since the B domain structure has not been experimentally solved in FV, nor in its homologue human Factor VIII, the structure prediction of the B domain is essentially arbitrary, even though this accounts for the presence of B domain in FV. The Ramachandran analysis showed that, for the full FV sequence of 2,224 residues, 71.6% (1592 residues) of amino acids were categorized in the “most favored” conformational regions, 12.1% (269) were in the “additional allowed” regions, and 16.3% (362) were conformational outliers. Overall, the quality of the AlphaFold FV structural model was comparable to those of the cryo-EM structures, with more conformational outliers present as the result of the disordered nature of the B domain. Regardless, the AlphaFold model is advantageous as it displays the whole FV protein structure. From this, the B domain was predicted to have a disordered outermost structure that was wrapped around the central core of the five A1-A3 and C1-C2 domains ( Figs. 3a,b ).

**Fig. 4:**
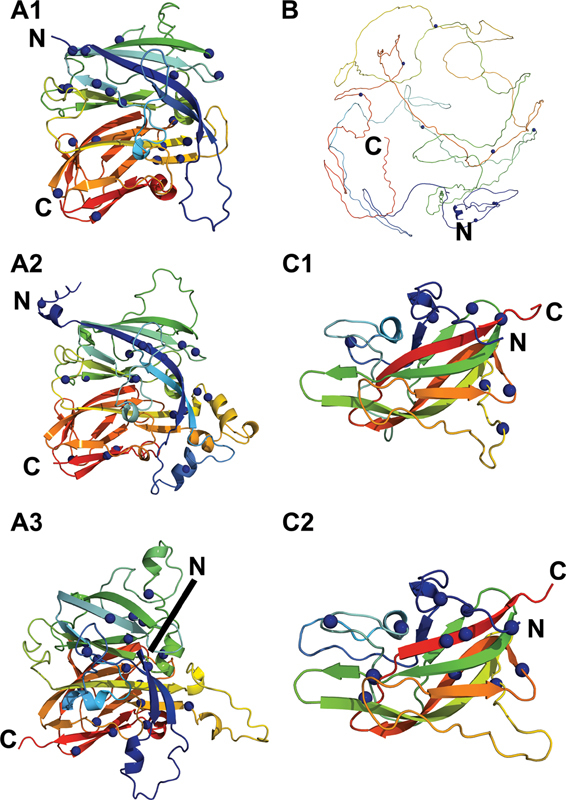
*The six individual FV domain structures and their 100 missense variants.* Each domain is shown as a ribbon diagram in rainbow colors from the N-terminus ( *blue* ) to the C-terminus ( *red* ) for clarity. The structurally similar A1, A2, and A3 domains are shown with their secondary structure ribbons depicted in the same orientations, and likewise the structurally similar C1 and C2 domains. The blue spheres denote the missense mutations associated with FV deficiency in each domain: A1, 22 variants; A2, 20 variants; A3, 22 variants; B, nine variants; C1, eight variants; C2, 17 variants. These variants total 98, where the remaining two variants (not shown) occur in the signal peptide (one variant) and the a2 linker (one variant). FV, Factor V.

The AlphaFold FV structure displays all 100 FV deficiency missense variants found in FV, these being color coded according to the phenotype for each variant ( Fig. 3c ). Green, yellow, and red spheres corresponded to mild, moderate, and severe cases, and were distributed throughout the six FV domains, similarly to the purple asymptomatic cases. These locations suggest that the whole FV structure is susceptible to the damaging effects of genetic variants, including the disordered B domain. In fact, the four most common missense variants ( Fig. 3d ), occurred in four different globular domains of FV, as well as the next 25 most common ones. Visualizing the missense variants for each individual domain demonstrated an even distribution of variants across all structures, including the disordered B domain. This outcome underscored the effect of the variants on disrupting the overall FV protein structure rather than disturbing localized functionally important regions ( Fig. 4 ).

**Fig. 5:**
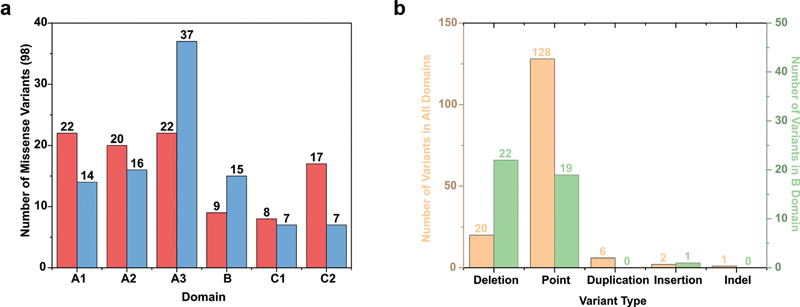
*Distribution of FV deficiency variants in the six FV domains* . ( **a** ) The number of missense variants in each of the six FV domains is shown above the red bars. If the number of 98 missense variants are normalized in proportion to amino acid residues present in the sequence of each domain, the outcome is shown as blue bars. The two missense variants that occur in the linker region and the signal peptide are not shown. ( **b** ) The distribution of 42 variants in the B domain ( *green* ) is compared against the five types of 157 genetic variants that occur in FV ( *orange* ). FV, Factor V.

Comparing the actual number of missense variants in each domain to the normalized number based on the size of each domain showed that slightly more variants occurred in most of the globular A and C domains and fewer in the A3 and disordered B domain ( Fig. 5 ). The percent differences between actual and normalized variants were 57, 25, and −68% in the A domains, and 14 and 43% in the C domains. The percent difference between actual and normalized variants in the B domain was −67%. The lower proportion of variants in the B domain was attributable to its disordered structure, where a residue change was less likely to perturb the B domain structure. In contrast, residue changes in the globular A and C domains were more likely to affect protein folding. Interestingly, the B domain showed more deletion variants occurring in this compared with the A and C domains ( Fig. 5b ). This could again be attributed to its disordered structure being more tolerant of traditionally highly damaging variants.

### Comparison of the Individual Variants with the FV Protein Structure

**Fig. 6:**
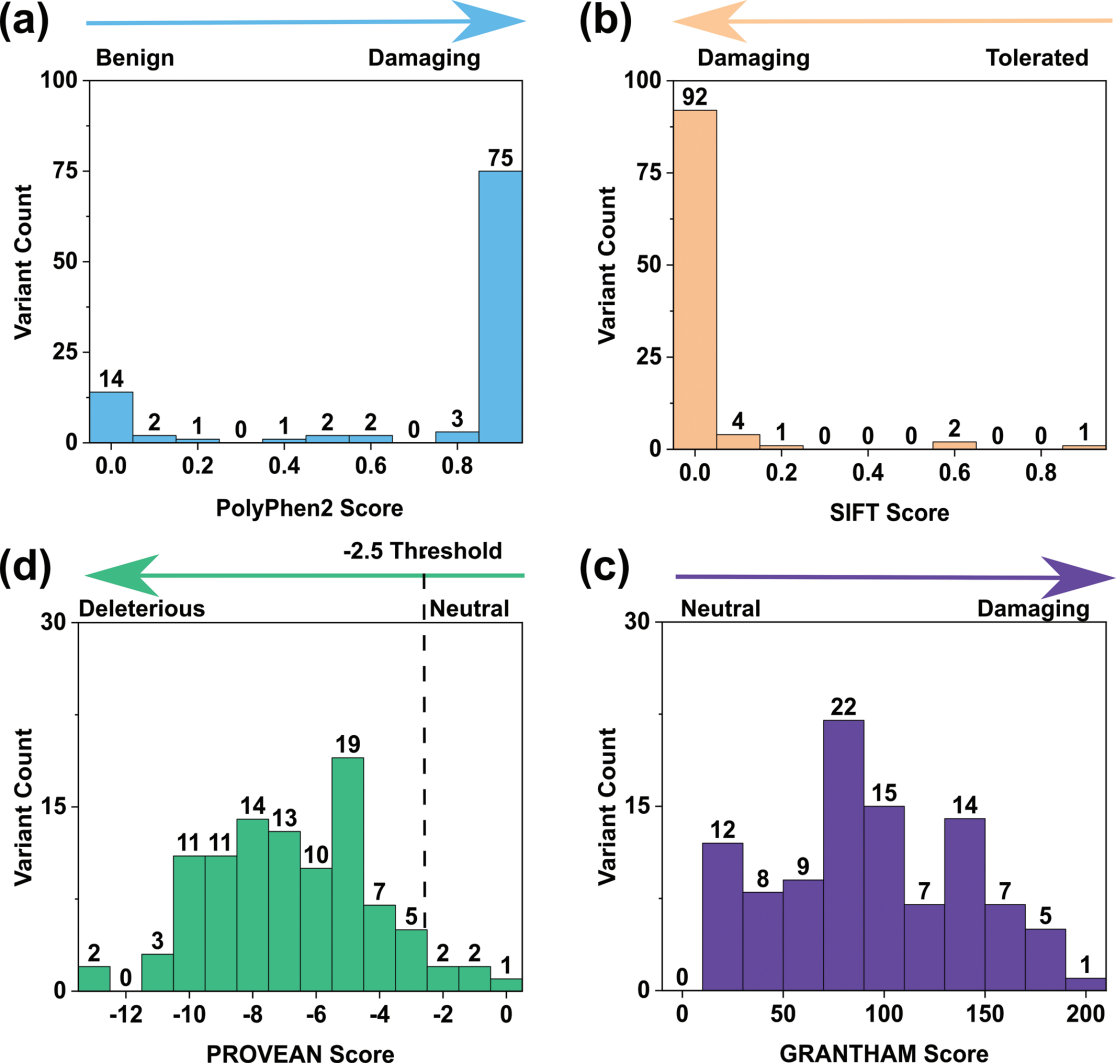
*Substitution analysis of 100 FV deficiency missense variants in the F5 gene.* The four substitution analyses predict the damaging effects of substitution variants on the protein structures. ( **a** ) Analysis of the variants determined by their PolyPhen-2 scores. ( **b** ) Analysis of variants determined by their SIFT scores. ( **c** ) Analysis of variants determined by their PROVEAN scores. The PROVEAN threshold used was −2.5. ( **d** ) Analysis of the variants determined by their Grantham scores. FV, Factor V.

The identification of a missense FV variant in a patient gene cannot be assumed to cause an FV disorder. Additionally, with 100 unique FV deficiency-associated missense variants, it was not feasible to experimentally test individual variants via recombinant expression to determine if the variant damages FV function or folding. To circumvent this issue, the FV website predicted whether an individual variant was likely to disrupt protein structure and function. Four independent algorithms were used. The PolyPhen-2 analysis predicted that, of the 100 missense variants, 75 (75%) gave scores between 0.9 and 1.0 and were assigned to be damaging, but that 25 (25%) were predicted to be benign variants ( Fig. 6a ). The SIFT analysis predicted that 92 (92%) of the missense variants were damaging ( Figs. 6b ). Therefore, most FV deficiency-associated missense variants are predicted to be damaging. The PROVEAN and Grantham analyses regarding potential damaging effects were less clear, with 95 (95%) PROVEAN and 88 (88%) GRANTHAM scores being deleterious in a wider distribution of scores. Regardless, both the PROVEAN and Grantham analyses also demonstrated that many variants were damaging ( Figs. 6c, d ). The interactive FV database provided all four PolyPhen-2, SIFT, PROVEAN, and Grantham scores for each of the 100 missense variants, to assist clinicians to make an informed decision about the significance of a given variant.

**Fig. 7:**
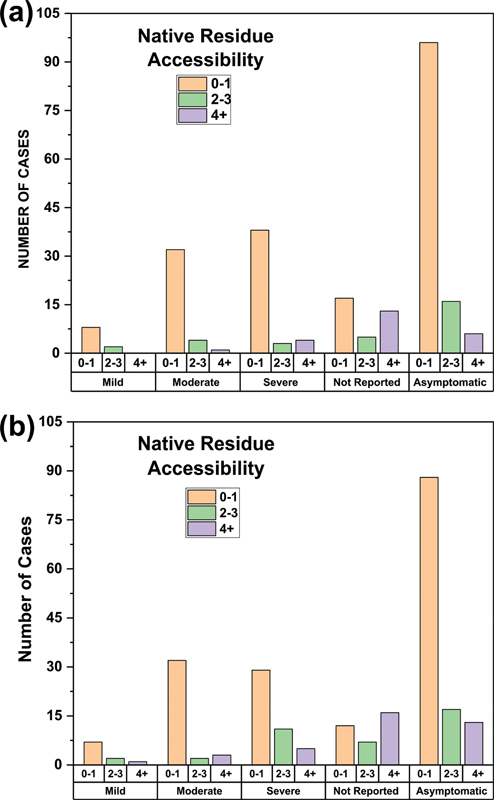
*Accessibility analyses of 100 missense variants associated with FV deficiency in the FV protein structure* . ( **a** ) Accessibilities are presented for the full protein. FV variants in the intact cryo-EM structure (PDB ID: 7KVE) and the full-length AlphaFold structure ( Supplementary Fig. S1 ) were grouped by their phenotypic classification (bottom row). The variants were further subdivided according to the native residue accessibility (accessibilities of 0–1, 2–3 and 4+) of the intact protein. The accessibility was determined using DSSP ( Supplementary Fig. S1 ). Accessibilities of 0 or 1 indicate side chain burial and values of >1 indicate side chain exposure to solvent. ( **b** ) Accessibilities are presented for the six separated domains. FV variants were again grouped by phenotypic classification and accessibility. Here, accessibility refers to the change in residue accessibility when the cryo-EM FV structure (PDB ID: 7KVE) or the AlphaFold structure ( Supplementary Fig. S1 ) was separated into the six A1, A2, A3, B, C1, and C2 domains. Interdomain linkers are not included in this comparison. FV, Factor V.

The relationship between FV phenotype and amino acid surface solvent accessibilities in the FV protein structure for each variant provided further information on the effect of each variant. Residue surface accessibilities were calculated using the DSSP tool for the intact FV protein and the six individually separated FV domains, then graphically compared with the reported phenotype for the variants. For the intact FV protein, 80% of the mild variants (8 of 10 cases) had low accessibilities of 0 or 1, indicating these were mostly buried within the FV structure ( Fig. 7a ). Similarly, for the moderate and severe phenotypes, 86% (32 of 37) and 84% (38 of 45) of the variants also had low accessibilities of 0 or 1. For the separated FV domains, the accessibilities showed a similar trend whereby 70% (7 of 10), 86% (32 of 37), and 64% (29 of 45) of the variants for the mild, moderate, and severe phenotypes had low accessibilities of 0 or 1 ( Fig. 7b ). The predominance of damaging variants at buried sites, such as those in interdomain contacts, illustrates that small perturbations in the FV structure were sufficient to reduce normal protein function and become disease associated.

The four most common missense variants ( Fig. 3d ) were visually highlighted to illustrate the effects caused by these amino acid changes ( Supplementary Fig. S3 ). The patient list shows that these four possessed a mix of all phenotypes. In keeping with Arg being the most commonly mutated residue in the FV variants ( Supplementary Fig. S2 ), one of the most common amino acid mutants was an Arg residue, with a Gly, Tyr, and Asp being the other three most common (Gly420, Tyr1730, Arg2102 and Asp96; HGVS), with accessibilities of 2, 0 and 1 and 0, respectively ( Supplementary Fig. S2 ). These occurred in 9 to 21 patients and were mutated to Cys, Cys, Cys and His residues ( Supplementary Fig. S3 ). The Tyr1730, Arg2102, and Asp96 (HGVS) variants were all buried within the globular FV structure as evidenced by the low accessibility scores of 0 or 1. Three variants were mutated to Cys residues, which will present an unpaired Cys residue that may interfere with the disulfide bridge pairings that stabilize the wildtype FV structure. The Asp96His (HGVS) mutation converts a negatively charged residue to a positive charge, most likely to interfere with critical internal stability contacts within FV, particularly between the A1 and C2 domains ( Supplementary Fig. S3(d) ). Gly420 (HGVS) has a higher accessibility score of 2; the visualization of this residue in Supplementary Fig. S4(a) supports this finding. In this case, the conversion of a positively charged residue to a polar uncharged residue may interfere with the stability of the A2 domain or a potential binding with the disordered B domain.

## Discussion

Here, our new interactive genetic and structural web database for human FV will facilitate improved analyses of FV genetic variants for clinicians, as well as further insights into the disease mechanism leading to FV deficiency. It was not feasible to analyze the FV Leiden variants here for reason of their reduced frequency. Given the high growth in newly reported FV variants, a simple flat listing of these is now overwhelming to use, and is unable to provide clinicians with useful information. Our interactive FV database is a powerful resource and has two predominant advantages: (1) the number of unique FV variants totals 363 variants, this being accumulated from the published literature and ClinVar ( Fig. 1 ); (2) the new cryo-EM molecular structures for FV and FVa 21 and the full-length AlphaFold prediction for FV, 20 21 enable the FV variants to be assessed in the context of the full three-dimensional structure of FV. The variants compiled in the database are distributed throughout the six globular and disordered FV domains. For all the missense variants, the PolyPhen-2, SIFT, PROVEAN, and Grantham analyses indicate that most variants are likely to be damaging to the FV protein. Interestingly, many detected variants are located in buried regions of low solvent accessibility ( Fig. 7 ). This outcome suggests that the alteration and perturbation of residues inside each FV domain are a major cause of FV deficiency due to disruption of the FV structure. While mutational hotspots in functionally important regions of FV can be important, these are not the dominant cause of FV genetic disease.

Several regions of FV/FVa are critical to its function and should be considered in the context of identified variants. Four major FVa ligands have been identified, namely FXa, thrombin, polyanionic membranes, and APC.

(1)First, we consider the FV regions involved in binding to FXa, labeled as BS1 to BS4 in Supplementary Fig. S4(a) in that order below. One possible site BS1 for FVa binding to FXa is the Arg334 region (legacy Arg306) in the A2 domain that involves either residues 339 to 353 in FV (legacy 311–325) 35 or a larger section in residues 335 to 376 (legacy 307–348). 36 In our dataset of 199 unique FV deficiency variants, mutations were found in 14% of the residues in the larger proposed binding region. Another region BS2 for the FXa binding site is the Arg534 region (legacy Arg506), with a binding cluster involving the residues Arg529, Arg534, Arg538, Ala539, Asp541, Asp605, and Asp606 (HGVS). 37 We identified variants only in residue Arg534, although several variants in close proximity to the binding residues were found. However, there is evidence that the Arg334 and Arg534 regions may bind prothrombin rather than FXa, or both, but this remains unclear. 38 A third FXa binding site BS3 has been suggested as the A2 domain C-terminus (A2T) from residues 711 to 737 (legacy 683–709). 39 We identified variants only in residue 717 in this binding region. A fourth region BS4 for FVa binding to FXa has been suggested in the A3 domain, spanning a large portion of the domain from residues 1,565 to 1,780 (legacy 1,537–1,752). 38 We identified variants in 10% of the amino acids in this binding region.
(2)Next, we consider FVa binding to prothrombin. The first binding region on FV for prothrombin is around regions Arg334 and Arg534 in the A2 domain ( Supplementary Fig. S4(a) ; sites BS1 and BS2), as mentioned above. The A2T binding site implicated in FXa binding also may play a role in binding prothrombin, with a variant at S717 being identified in our database. However, contradictory evidence exists with regard to the validity of this region in binding prothrombin. The light chain of FV (domains A3, C1 and C2) does not appear to be involved in prothrombin binding. 38
(3)The third key interaction is the binding of FVa to anionic membranes (sites BS1 and BS2 in Supplementary Fig. S4(a) ), which is critical for formation of the ternary prothrombinase–prothrombin complex involving FVa, FXa, and prothrombin. Residues 1982 to 1985, 2051, and 2055 (legacy 1954–1957, 2023, and 2027) in the C1 domain form hydrophobic “spikes” which help anchor FVa to the anionic membrane. 38 We did not identify any variants in this binding region. The C2 domain also plays a role in binding anionic membranes, with the N-terminal region from 2065 to 2115 (legacy 2037–2087) containing hydrophobic and electrostatic interactions which aid binding. 40 We identified variants in 18% of the residues in this region.
(4)A final critical binding interaction is APC binding to FVa to inactivate it and regulate the coagulation process. The binding site for APC to FVa is not known. 41 However, it is well-established that APC cleaves FV at R334, R534, and R707 (legacy R306, R506, and R679). We identified variants in residue 534, indicating the importance of this residues for proper regulation ( Supplementary Fig. S4 ).

In summary from the above, variants have been identified in up to 18% of the residues comprising each of these six functionally important regions. Overall, for the 185 unique variants found in the exonic region of *F5* , only 38 or 20% corresponds to variants in any of the functional regions of FV. These functionally important regions comprise 15% of the total number of 2,224 residues in FV. This outcome indicates that 80% of identified variants are found in the remaining 85% residues of FV which lie outside the functional regions. It is concluded that mutation rates were not higher in the functional portions of FV. Interestingly, for the four most commonly mutated residues according to number of patients (Asp96, Gly420, Tyr1730, and Arg2102 [HGVS]), two occur within functional regions of FV. Overall, this distribution of variants in FV in both the functional regions and other regions distant from functional areas shows that both are capable of disrupting the overall FV structure and causing disease. Therefore, FV function is susceptible to disturbances in both its globular and disordered structure.

The majority of variants in our database were associated with FV deficiency, and these variants were found in the full sequence of residues 15 to 2,222 in FV. Therefore, FV deficiency is associated with variants found across the length of FV rather than just in functionally active regions. The changes to protein structure due to these variants inactivate FV, preventing conversion of prothrombin to thrombin and increasing the risk of bleeding. The database is able to assess whether a variant is significant in terms of its potential effect on the protein structure from the four scoring methods and its solvent accessibility ( Supplementary Fig. S5 ). While not analyzed in detail, FV Leiden or thrombosis was also associated with variants that occur across the full length of the FV sequence.

FV has a unique protein domain structure in comparison to most of the coagulation proteins. It features globular A and C domains in a compact arrangement, these being surrounded by a disordered B domain ( Fig. 3 ). FV shares a similar domain structure with Factor VIII. Other coagulation proteins vary in their three-dimensional structure. For example, Factor XI possesses a compact five-domain globular structure whereas Factors IX and X have extended four-domain structures. Regardless of these structural differences, variants were distributed throughout each of these proteins, suggesting these variants disrupt the protein folding, thereby damaging function. The same tendency for variants to be distributed across the domain structures rather than localized to functional hotspots was present in these different proteins. For FV, the globular (A1-A3, C1-C2) and disordered (B) domains represent 58 and 35% of the 2,224 residues in its sequence, respectively, and possess 65 and 21% of FV deficiency variants in the protein. The evidence suggests FV function is susceptible to perturbations in its structure regardless of the location of a variant, even in the disordered B domain. It had been suggested that most missense variants in the B domain of Factor VIII are unlikely to be disease-associated as the B domain is unnecessary for secretion and function of the protein. 42 Although the B domains of FV and Factor VIII do not show high sequence similarity, there is not enough evidence to show whether variants in the B domains of FV or Factor VIII are more or less likely to be causative of disease.

Following the established format of our FXI, 13 32 FIX, 23 and FX 14 web databases, the FV website layout presents separate genetic and structural information in parallel with each other. The database will facilitate additional research into FV to understand better the relationship between FV deficiency and disease severity, based on the substantial number of FV variants and their new structural interpretation afforded by the cryo-EM structures and the AlphaFold prediction. Variants predicted to be damaging to the FV structure can be prioritized for experimental analysis to understand better how the structure is disrupted.

## Notes

**What Is Known on This Topic**

- A large number of FV variants have been published in the literature and resources such as ClinVar, however, no interactive web database dedicated to FV has been available.
- New structural models for FV now include the disordered B domain and recent cryo-EM structures for the A1-A2-A3-C1-C2 domains, thus enabling an assessment of the FV missense variants in terms of the full FV protein structure compared with earlier.

**What This Paper Adds**

- As a clinical research tool, we summarize 363 unique FV variants from 801 case reports. For FV deficiency, 199 variants were associated with FV deficiency from 245 patient records, in our interactive web database, for which there are 26 mild, 22 moderate, 49 severe, 35 asymptomatic, and 67 unreported phenotypes. For the risk factor FV Leiden, 37 unique variants were reported.
- Variants were distributed evenly throughout the globular (A1-A3, C1 and C2) and disordered (B) domains without the evidence of localized hotspots. The globular and disordered domains comprise 58 and 35% of the full FV sequence, respectively, and contain 64 and 21% of the variants.
- The frequency of FV variants across its sequence suggests that FV deficiency results from the sensitivity of FV function to protein structural disruption, rather than the inhibition of its binding to other proteins.
