# Supplementary material for: Analysis of 363 Genetic Variants in F5 via an Interactive Web Database Reveals New Insights into FV Deficiency and FV Leiden
Source: TH Open. 2023 Jan 9;7(1):e30–41. doi: 10.1055/a-1987-5978 (PMC9829979; doi:10.1055/a-1987-5978)
Supplement: Supplementary file 1 — Supplementary Material [file 10-1055-a-1987-5978-s22080039.pdf]

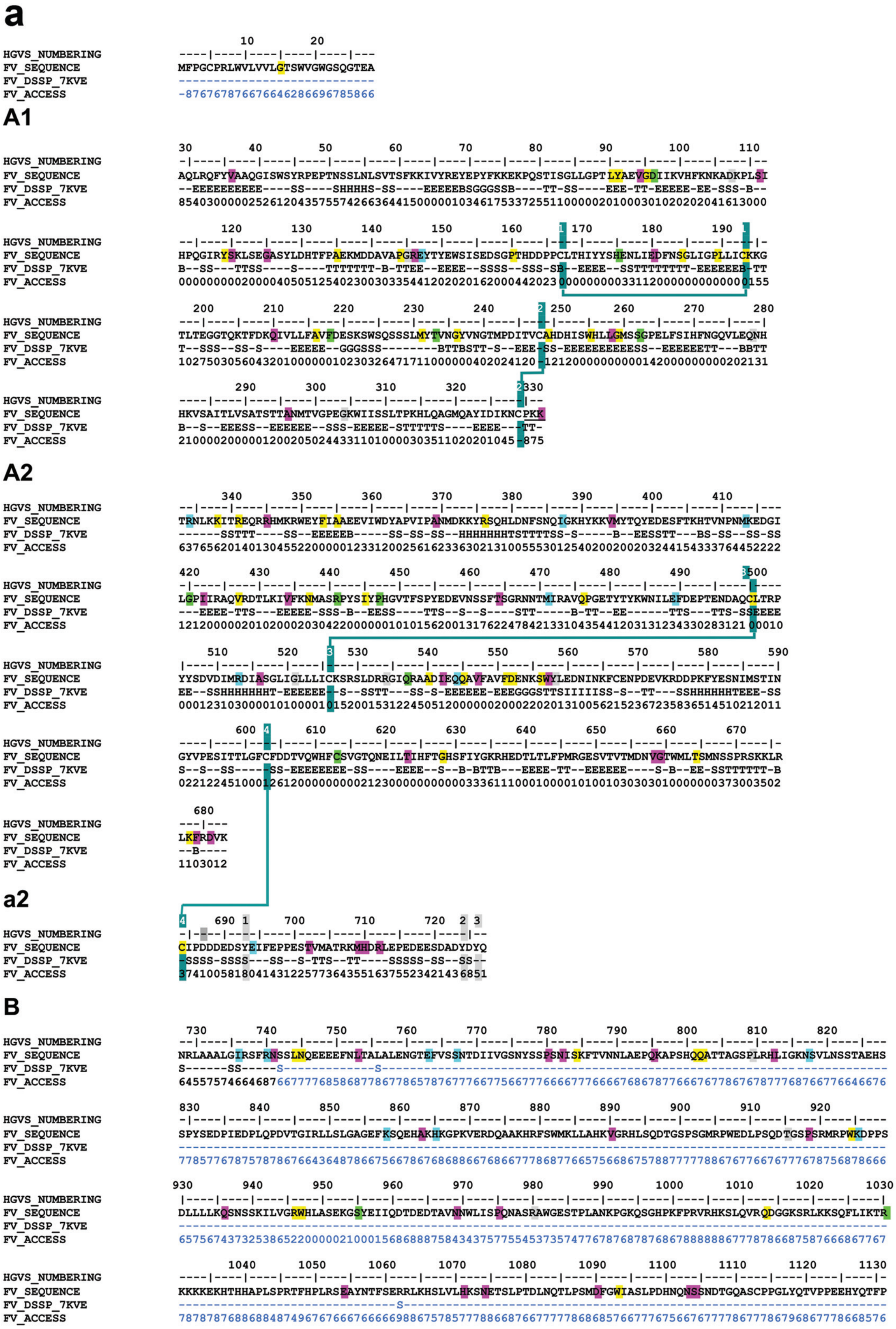

Supplementary Fig. S1 (**a, b**) *Secondary structure and accessibility analysis of variants occurring in the FV protein.* The FV amino acid sequence is shown with secondary structure assignments and solvent accessibilities indicated below each residue. The residue secondary structures are denoted as either H (α-helix), B (β-bridge), E (extended β-strand), G (3_{10} helix), I (π-helix), T (hydrogen-bonded turn), S (bend), or C (undefined coil region). Secondary structures and accessibilities based on a defined cryo-EM structure are marked in black, and those without a cryo-EM structure are marked in blue. For these latter residues, secondary structure and solvent accessibility predictions were made based on the AlphaFold structure. The positions of 285 point variants that occur in the exonic region of the *F5* gene are highlighted in boxes. These include point missense, point nonsense, and point silent variants. Yellow boxes denote point variants that occur in four patients or less, green boxes denote point variants that occur in five patients or more, and red boxes denote point variants that occur in over 50 patients. Post-transcriptional modifications are shown. These include seven Cys-Cys disulfide bridges (*highlighted in blue*) and seven sulphated Tyr residues (*highlighted in gray*). FV, Factor V.

 © 2023. The Author(s).

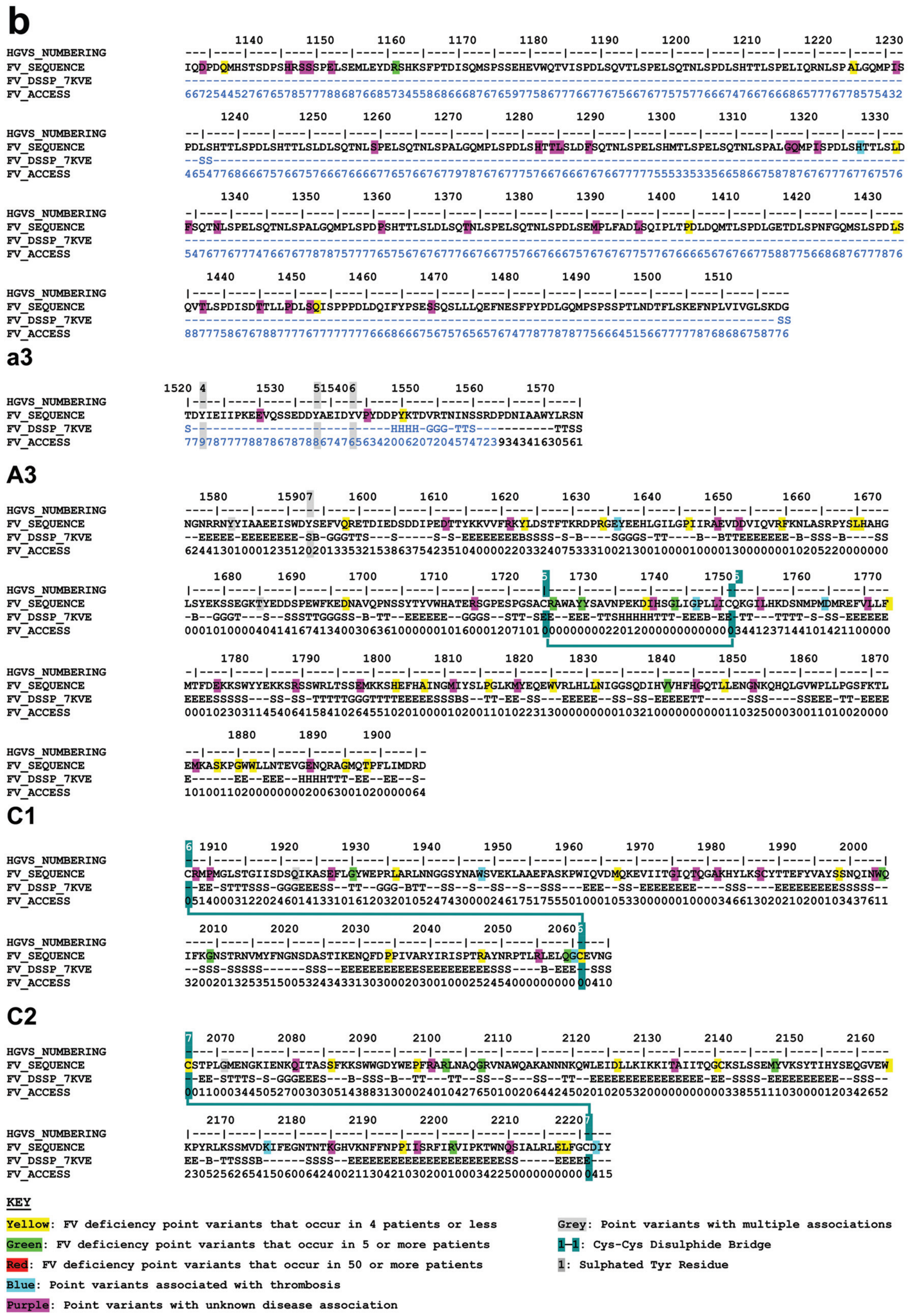
b
a3
A3
C1
C2
HGVS_NUMBERING
FV_SEQUENCE
FV_DSSP_7KVE
FV_ACCESS
KEY
Yellow: FV deficiency point variants that occur in 4 patients or less
Green: FV deficiency point variants that occur in 5 or more patients
Red: FV deficiency point variants that occur in 50 or more patients
Blue: Point variants associated with thrombosis
Purple: Point variants with unknown disease association
Grey: Point variants with multiple associations
1–1: Cys-Cys Disulphide Bridge
1: Sulphated Tyr Residue

AA MUTATED TO (rows: **AA MUTATED FROM**)

	Ala	Arg	Asn	Asp	Cys	Gln	Glu	Gly	His	Ile	Leu	Lys	Met	Phe	Pro	Ser	Thr	Trp	Tyr	Val	SUM
Ala				0			0	0							1	0	1			0	2
Arg					1	1		4	2	0	2	0	1		4	0	1	2			18
Asn				0					0	0		0				1	0		2		3
Asp	2		1			0	0	3	0										1	0	7
Cys		5						1						0		0		1	2		9
Gln		0					0		0		0	0			0						0
Glu	0			0		0		3				1								0	4
Gly	0	0		2	1		0									1		0		0	4
His		1	0	2		1					0				0				0		4
Ile		1	0								0	0	0	0		1	0			0	2
Leu		1				0			0	0			0	1	3	1		0		0	6
Lys		0	0			0	1			0			1				0				2
Met		0								0	0	0					1			1	2
Phe					0					0	2					0			0	0	2
Pro	0	1				1			0		3					1	0				6
Ser	0	1	1		1			1		0	2			2	2		0	0	1		11
Thr	1	0	0							1		0	1		1	0					4
Trp		1			0			0			0					1					2
Tyr			0	2	3				0					1		0					6
Val	3			0			0	3		0	0		0	0							6
SUM	6	11	2	6	6	3	1	15	2	1	9	1	3	4	11	6	3	3	6	1	100

Supplementary Fig. S2 *Substitution grid representing 100 point missense FV deficiency variants in the F5 gene.* The grid presents the number of missense variants that occurs for each defined amino acid change. All the substitutions are the result of a single nucleotide change. Any grid substitution that would require more than a single nucleotide change is shown in dark gray, however, none were seen. Silent variants are excluded from the grid and shown in pale gray. White boxes represent possible substitutions that do not occur in the *F5* gene. Yellow boxes represent substitutions that occur between one and five times. FV, Factor V.

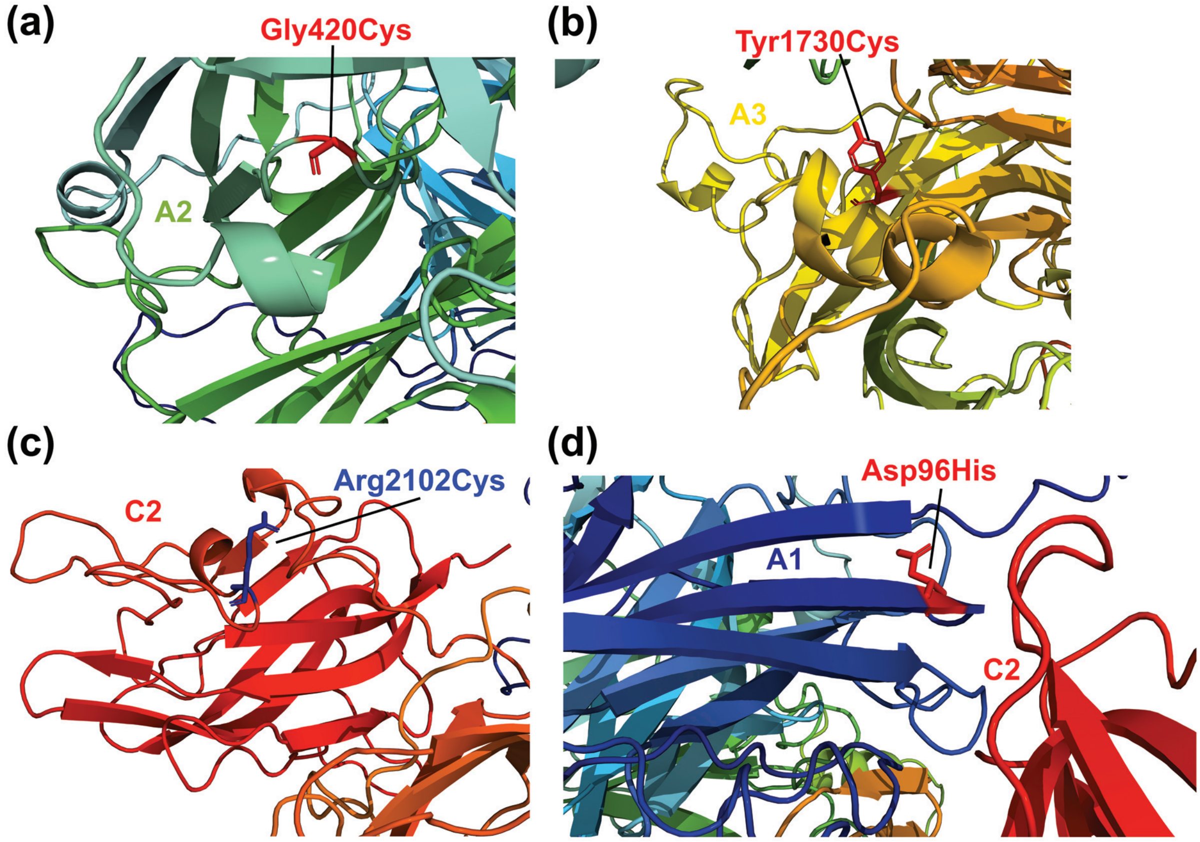

Supplementary Fig. S3 *Molecular graphic representation of four residues within the FVa protein structure.* The four panels highlight in the FVa cryo-EM structure the four most-commonly occurring variants found in patients (►**Fig. 3d**). For each, the residue of interest in the native FVa protein is shown. Panels (**a–d**) highlight the wildtype residues Gly420 (A1 domain; 10 patients), Tyr1730 (A3 domain; 21 patients), Arg2102 (C2 domain; 9 patients), and Asp96 (A1 domain; 11 patients). All residue numbering is given in HGVS format. FV, Factor V.

 © 2023. The Author(s).

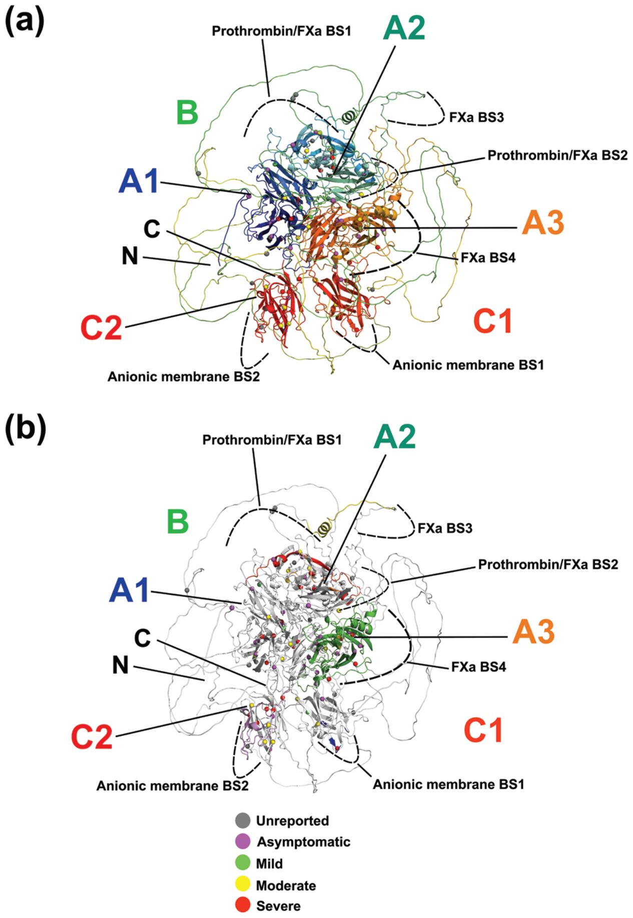

Supplementary Fig. S4 *FV protein structure with labeled FV deficiency missense variants and known protein contact regions.* (**a**) The full FV structure is shown in ribbon format from the AlphaFold prediction using artificial intelligence. The structure is shown in rainbow colors, with blue corresponding to the N-terminal region and red corresponding to the C-terminal region. The N-terminus and C-terminus are denoted by N and C, respectively. The 100 missense variants are mapped to the ribbon diagram, where the phenotype classifications of mild, moderate, and severe effects are denoted as the traffic light colors of green, yellow, and red. Missense variants with unreported phenotype are shown in gray and asymptomatic cases are shown in purple. The dotted lines denote the regions of FV (FVa) that interact with coagulation proteins FXa and prothrombin, as well as the anionic phospholipid cell membrane. Multiple binding sites (BS) for the same factor are numbered accordingly. Note that the APC binding site is not indicated as it is not known. (**b**) The full FV structure is shown in the same format as (**a**) except the main chain has been colored gray and the binding sites were colored red, orange, yellow, green, blue, and violet to highlight these better. FV, Factor V.

 © 2023. The Author(s).

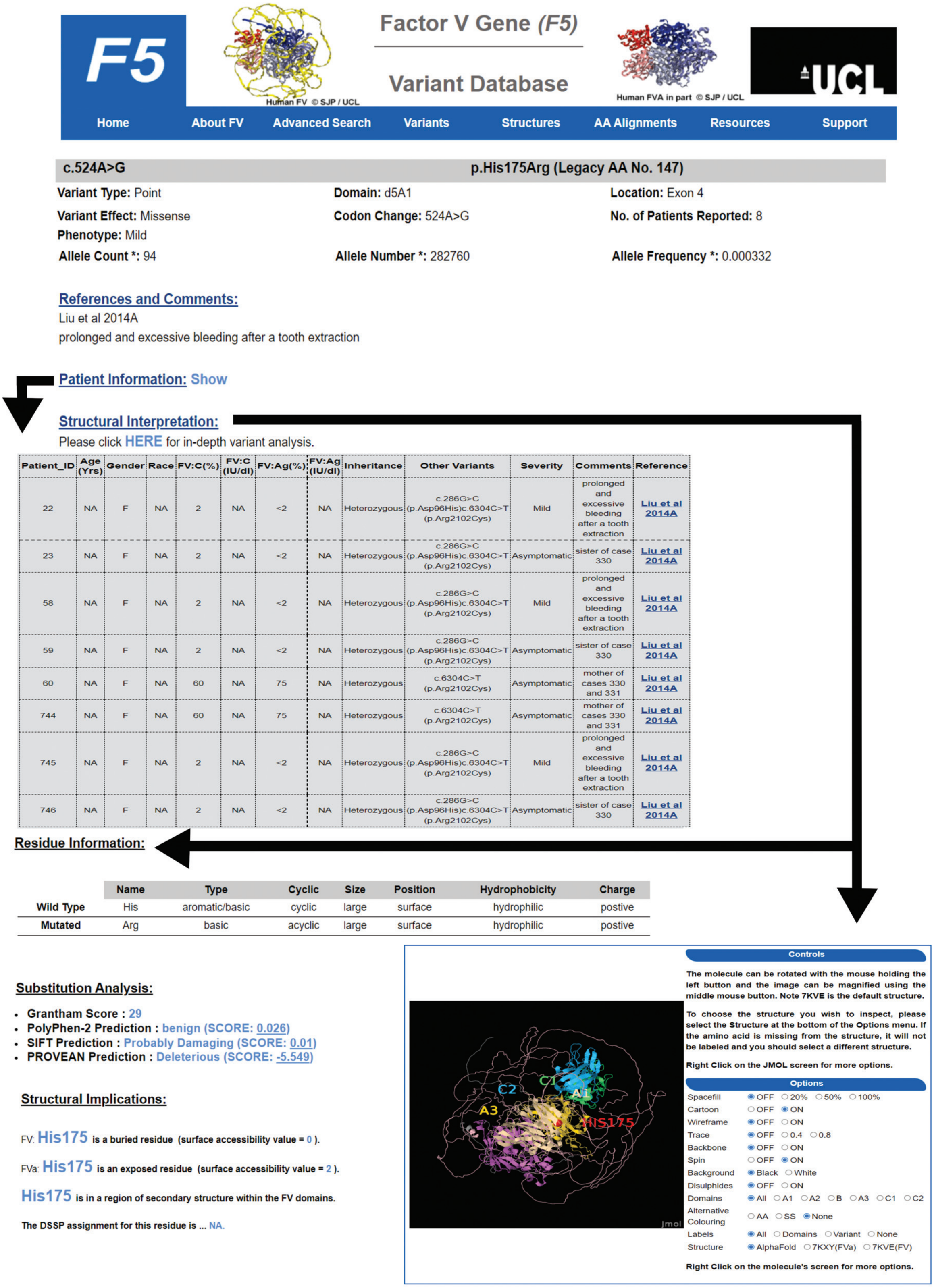

Supplementary Fig. S5 *Screenshots of the FV website to illustrate the analysis made for the His175Arg variant.* The upper panel displays the output when the His175 residue is inputted on the home page of the interactive website. By clicking "Show" on the patient information, the lower left panel lists genetic information for the eight patients reported with the His175Arg variant together with the source of the patient record. Clicking "HERE" on the structural interpretation gives the image shown on the bottom right panel. This assesses the buried or exposed accessibility of the variant and its location in the FV protein structure. A JMol view of the FV structure is displayed that can be rotated and zoomed into as desired; users also can switch between FV structures. The Grantham, Polyphen-2, SIFT, and PROVEAN substitution analyses to predict the damaging effects of each missense variant are provided to facilitate clinical diagnosis. FV, Factor V.

 © 2023. The Author(s).
